# Heart‐Specific Overexpression of Choline Acetyltransferase Gene Protects Murine Heart Against Ischemia Through Hypoxia‐Inducible Factor‐1α–Related Defense Mechanisms

**DOI:** 10.1161/JAHA.112.004887

**Published:** 2013-02-22

**Authors:** Yoshihiko Kakinuma, Masayuki Tsuda, Kayo Okazaki, Tsuyoshi Akiyama, Mikihiko Arikawa, Tatsuya Noguchi, Takayuki Sato

**Affiliations:** 1 Department of Cardiovascular Control, Kochi Medical School, Nankoku, Japan (Y.K., K.O., M.A., T.N., T.S.); 2 Institute for Laboratory Animal Research, Kochi Medical School, Nankoku, Japan (M.T.); 3 Department of Cardiac Physiology, National Cardiovascular Center Research Institute, Suita, Osaka, Japan (T.A.)

**Keywords:** acetylcholine, choline acetyltransferase, energy metabolism, hypoxia‐inducible factor

## Abstract

**Background:**

Murine and human ventricular cardiomyocytes rich in acetylcholine (Ach) receptors are poorly innervated by the vagus, compared with whole ventricular innervation by the adrenergic nerve. However, vagal nerve stimulation produces a favorable outcome even in the murine heart, despite relatively low ventricular cholinergic nerve density. Such a mismatch and missing link suggest the existence of a nonneuronal cholinergic system in ventricular myocardium.

**Methods and Results:**

To examine the role of the nonneuronal cardiac cholinergic system, we generated choline acetyltransferase (ChAT)–expressing cells and heart‐specific ChAT transgenic (ChAT‐tg) mice. Compared with cardiomyocytes of wild‐type (WT) mice, those of the ChAT‐tg mice had high levels of ACh and hypoxia‐inducible factor (HIF)‐1α protein and augmented glucose uptake. These phenotypes were also reproduced by ChAT‐overexpressing cells, which utilized oxygen less. Before myocardial infarction (MI), the WT and ChAT‐tg mice showed similar hemodynamics; after MI, however, the ChAT‐tg mice had better survival than did the WT mice. In the ChAT‐tg hearts, accelerated angiogenesis at the ischemic area, and accentuated glucose utilization prevented post‐MI remodeling. The ChAT‐tg heart was more resistant to ischemia–reperfusion injury than was the WT heart.

**Conclusions:**

These results suggest that the activated cardiac ACh‐HIF‐1α cascade improves survival after MI. We conclude that de novo synthesis of ACh in cardiomyocytes is a pivotal mechanism for self‐defense against ischemia.

## Introduction

The heart, especially the left ventricle, is predominantly innervated by adrenergic nerves, whose nerve endings are distributed in both ventricles. This is in great contrast to cholinergic nerve endings, whose density is substantially lower than that of the adrenergic nerve ending, and the density is exclusively higher in the atria and the specialized conduction system. The biological significance of acetylcholine (ACh) is well known; for example, ventricular contractility of the heart paced at a constant rate is decreased by ACh infusion into coronary arteries but not by vagal nerve stimulation (VNS), suggesting that ACh released from the nerve endings by VNS may not be responsible for the negative inotropic effect. Despite the biological significance of ACh, its source in ventricular myocardium remains unclear.

Recently, we showed a novel de novo ACh‐synthesis system in the heart, because cardiomyocytes possess the capacity to enzymatically synthesize and store ACh, and we suggested the biological significance of the system in cardiomyocytes from the viewpoint of energy metabolism. Specifically, the de novo ACh‐synthesis system, that is, the nonneuronal cardiac cholinergic system, modulates mitochondrial function to regulate the metabolic rate^1–2^; moreover, ACh synthesis is transcriptionally activated through a muscarinic receptor in a positive‐feedback loop.^1^ This strongly supports that a muscarinic receptor is a prerequisite for cardiomyocytes synthesizing ACh. This nonneuronal cardiac cholinergic system, later independently reported by Rana et al,^3^ is expected to close the gap between our results of the beneficial effects of chronic vagal nerve stimulation on chronic heart failure^4–9^ and the previous findings that the cardiac ventricles are sparsely innervated by the vagus compared with adrenergic nerves.

To clarify the role of the nonneuronal cardiac cholinergic system in diseased hearts, we developed a heart‐specific choline acetyltransferase transgenic (ChAT‐tg) mouse, and subjected it to myocardial infarction (MI) and ischemia–reperfusion. The present results shown here suggest that cardiomyocyte‐derived ACh plays a pivotal role in modulating myocardial energy metabolism, for example, activated myocardial glucose utilization, and angiogenesis in ischemic areas after MI and that overexpression of cardiomyocyte ChAT improves survival after MI through antiremodeling effects or ischemia–reperfusion injury. The cardiac cholinergic system would be a novel therapeutic target against myocardial ischemia and chronic heart failure.

## Methods

### Generation and Identification of Transgenic Mice

An α‐MHC promoter region to specifically overexpress murine choline acetyltransferase (ChAT) in the heart was used to develop the ChAT‐tg mouse.^10^ Specific primers were used to subclone the mouse *ChAT* transgene, that is, a 1938‐bp cDNA encoding the entire open‐reading frame amplified by polymerase chain reaction into a transgene plasmid cassette containing a 5.5‐kb α‐MHC promoter, which drove not only the ChAT transgene but also the internal ribosome entry site (IRES)–regulated enhanced GFP (*EGFP*) gene. The transgene plasmid cassette was prepared by reference to the backbone plasmid construct of a pIRES‐EGFP vector (Clontech Laboratories, Mountain View, CA). This construct was then linearized by double digestion with *Nhe*I and *Ssp*I and purified before pronuclear injection was performed at the Institute for Laboratory Animal Research, Kochi University (Nankoku, Japan). The genetic background of the mouse strain used was C57BL/6. Originally, 2 founder lines were established. Litter sizes and postnatal development were indistinguishable from those of nontransgenic littermate controls. Specific primers flanking the EGFP coding sequence or flanking between the murine *ChAT* transgene and α‐MHC promoter sequence were used to screen offspring by genomic DNA.

### Animals

All animal procedures were performed using 60 male WT (C57BL/6) mice (Japan SLC, Inc, Hamamatsu, Japan) and 62 ChAT‐tg mice aged between 10 and 12 weeks. These procedures were in strict accordance with the recommendations in the guidelines of the Physiological Society of Japan, and the protocols were approved by the Animal Research Committee of Kochi Medical School (Permit No. E‐00017). The surgical procedures were performed under sodium pentobarbital anesthesia, and all efforts were taken to minimize suffering.

### Cell Culture and Transfection

Human embryonic kidney 293 (HEK293) cells were cultured in the DMEM culture medium containing 1000 mg/L glucose (low‐glucose medium), 10% FBS, and antibiotics. Subconfluent HEK293 cells (50% to 70%) were transiently transfected with murine ChAT overexpression vector using Effectene cationic transfection reagent (QIAGEN, Hamburg, Germany). A pIRES‐EGFP expression vector was used to develop the ChAT expression vector, into which a mouse ChAT full‐coding sequence was subcloned. As previously reported,^6^ a fluorescent microscope was used to easily identify the ChAT‐transfected cells, which showed an average transfection rate of 80%.

The double‐stranded ChAT‐specific Block‐iT miR RNA interference Select oligonucleotides (Life Technologies, Carlsbad, CA) were subcloned into miRNA expression vectors (pcDNA 6.2‐GW⁄ EmGFP‐miR; Life Technologies Japan) for preparation of ChAT‐knockout (KO) cells. ChAT‐specific oligonucleotides have already been verified for silencing *ChAT* gene expression.^1^ The vector‐transfected HEK293 (ChAT‐KO) cells created through the usage of Effectene were incubated with a selection of antibiotic blasticidin (10 μg/mL). The stable cell transfectants were then checked for the suppression level of ChAT expression. As a negative control, miRNA expression vectors with LacZ sequences were also developed (LacZ‐KO cells).

Neonatal hearts from the WT and ChAT‐tg mice were used to prepare primary cultured cardiomyocytes. The hearts were excised from 2‐ to 3‐day‐old neonates and digested for 30 minutes at 37°C in a mixture of 0.05% of collagenase types II and IV with agitation. This procedure was repeated twice; the digested hearts were then filtered and cultured in DMEM/Ham's F‐12 that included 10% FBS and ITS‐X (Life Technologies) on gelatin‐coated multiwell culture dishes.^11^ Five days after initiation of the culture of cardiomyocytes, they were exposed to 1% hypoxic conditions for 24 hours, followed by incubation under normoxic conditions for 2 hours until sampling.

Likewise, rat cardiomyocytes were also isolated for primary culture according to the procedure detailed in our previous study,^11^ and they were transiently transfected by the murine ChAT overexpression vector using Effectene cationic transfection reagent (QIAGEN) or by commercially verified rat ChAT‐specific siRNA oligonucleotides for gene silencing (QIAGEN) using a HiPerFect transfection reagent.

### Western Blot Analysis

HEK293 cells were transiently transfected with ChAT expression vectors for 3 days, and the cell lysates were then prepared. Samples from tissues were homogenized in T‐PER homogenizing buffer (Thermo Scientific, Rockford, IL) followed by centrifugation, and the supernatant, the protein concentration of which was quantified for comparable application, was used as a lysate for the further study. Cell lysates were mixed with a sample buffer, fractionated by 7.5% to 15% SDS‐PAGE, and transferred onto polyvinylidene fluoride membranes (Merck Millipore, Billerica, MA). The membranes were incubated with the following primary antibodies: (1) monoclonal antibodies against HIF‐1α (Novus Biologicals, Littleton, CO), phospho‐Akt and Akt (Cell Signaling Technology, Danvers, MA), and α‐tubulin (Lab Vision, Fermont, CA); (2) polyclonal antibodies against connexin‐43 (Zymed Laboratories, South San Francisco, CA), ChAT (Merck Millipore), GLUT‐1 and ‐4 (Merck Millipore), Akt (Cell Signaling Technology), β‐catenin (Cell Signaling Technology), and GFP (Medical & Biological Laboratories, Nagoya, Japan). They were then reacted with an HRP‐conjugated secondary antibody (BD Transduction Laboratories, San Diego, CA, and Promega, Madison, WI). An enhanced chemiluminescence system (Merck Millipore) was used to detect positive signals. In each study, these experiments were repeated 3 to 4 times. Only representative data alone are shown. The similarity of the sample loading volumes was further confirmed by expression of α‐tubulin or Coomassie brilliant blue staining. Representative blot data are shown from independently performed experiments (n=4 to 8).

### MTT Reduction Activity Assay

To evaluate the effects of ChAT overexpression on the mitochondrial function of primary cultured cardiomyocytes, we measured the reduction activity of 3‐(4,5‐dimethylthiazol‐2‐yl)‐2,5‐diphenyl tetrazolium bromide (MTT) in cardiomyocytes from the WT and ChAT‐tg mice. Five days after inoculation, when the cardiomyocytes started to beat in a synchronized manner, they were treated with an MTT reagent (Nacalai Tesque, Kyoto, Japan); it was added to the culture media, and the cardiomyocytes were incubated for 2 hours. After formazan was developed with purple crystal formation, isopropyl alcohol was added, and the absorbance at 570 nm was measured. As previously reported, MTT reduction activity depends on cellular metabolism, and even with comparable cell numbers, MTT activity decreases with decreased energy metabolism.^2,12^ Therefore, in this study, we also recognized the MTT activity as a marker of energy metabolism because cells were neither dead nor lost during the experiments.

### ATP Measurement

ATP levels in cells and tissues were evaluated using an XL‐ATP kit (APRO Life Science Institute, Naruto, Japan) according to the manufacturer's protocol. The ATP levels were adjusted for the protein levels.

### HPLC for ACh Assay

According to our previous study, we measured ACh contents in the heart by high‐performance liquid chromatography (HPLC).^1^ Briefly, the whole heart was excised, the atria were immediately removed from the heart, and the ventricles were immediately transferred into 0.1 mol/L of perchloric acid including 0.1 mmol/L of physostigmine and an internal control of isopropylhomocholine (5×10^−7^ mol/L). And then they were homogenized, centrifuged, and filtrated to obtain an HPLC sample. A quantitative analysis of ACh was performed by HPLC. The sample (10 μL) was injected into an HPLC system (HTEC‐500; Eicom, Kyoto, Japan). Peak data were recorded and analyzed with a computer.

### Immunohistochemical Study

The heart was excised, fixed in 4% paraformaldehyde, routinely processed, paraffin‐embedded, and cut into 4‐μm sections. Some hearts were processed with Masson's trichrome stain. Other samples were used for an immunohistochemical study using a Ventana automated immunohistochemistry system (Discovery, Ventana Medical System, Tucson, AZ). Antigen retrieval was performed for 60 minutes in a microwave‐preheated Dako Target Retrieval Solution (pH 6.0; Dako, Glostrup, Denmark), followed by inhibition of intrinsic peroxidase, blocking, and reaction with a primary antibody. Polyclonal antibodies against Glut‐1, Glut‐4 (both at 1:100; Merck Millipore), and von Willebrand factor (at 1:100; Dako) were used to identify immunoreactivities against Glut‐1, Glut‐4, and von Willebrand factor, respectively, on the basis of the streptavidin–biotin–peroxidase reaction.

### Echocardiographic and Hemodynamic Analysis

Heart rate (HR) and systolic (SBP) and diastolic (DBP) blood pressure were measured noninvasively in conscious mice by a tail cuff method. Mice were prewarmed at 37°C until measurement, and then a noninvasive BP measurement device (Softron, Tokyo, Japan) was used to perform hemodynamic measurements. For each mouse, 5 consecutive measurements were performed to allow averaging of the data.

For the conventional echocardiographic study, mice were anesthetized with 18 mg/kg of pentobarbital administered intraperitoneally. This dose was previously identified as having a lesser effect on cardiac performance compared with those of other anesthetics and gave sustained HRs from 450 to 550 bpm.^13–14^ A Vevo 2100 imaging system (VisualSonic, Toronto, Ontario, Canada) with a 30‐MHz scan probe was used to obtain 2‐dimensional transthoracic echocardiograms in the WT and ChAT‐tg mice. Two‐dimensional parasternal long‐axis imaging was used as a guide to obtain left ventricular M‐mode tracings. The end‐diastolic interventricular septum (IVSTd), left ventricular posterior wall thickness (LVPWd), and left ventricular diastolic (LVDd) or systolic internal (LVDs) diameters were measured. Ejection fraction (EF) and fractional shortening (FS) were calculated according to standard formulas.

### Myocardial Infarction

MI was developed by ligation of the left anterior descending coronary artery (LAD). Under anesthesia with pentobarbital (30 mg/kg IP) followed by artificial ventilation, a small left thoracotomy was performed to easily detect the LAD from the aorta. After ligation with a surgical suture (8‐0 silk suture), the hearts were replaced, and the thoracotomy wound was closed.^15^ Fourteen days after LAD ligation, the mice with MIs were sacrificed.

### Glucose Tolerance Test

d‐Glucose (1 mol/L) was intraperitoneally administered with an appropriate volume (0.2 to 0.3 mL), and an Accu‐Check active II (Roche Diagnostic, Tokyo, Japan) was used to evaluate blood glucose concentrations in blood collected sequentially at 0, 15, 30, 60, and 90 minutes.

### Ischemia–Reperfusion Injury Using a Langendorff Apparatus

After anesthesia with pentobarbital, the heart was excised from a male mouse (WT or ChAT‐tg) and was immediately connected to a Langendorff apparatus. The heart was perfused with filtered Krebs–Henseleit buffer at 80 to 85 mm Hg, equilibrated with 5% carbon dioxide and 95% oxygen. After stabilization, the heart was subjected to 30 minutes of global ischemia by stopping the buffer perfusion, followed by 60 minutes of reperfusion. The interval from ischemia to ventricular beating arrest due to ischemic contracture and the interval from the onset of reperfusion to recovery of ventricular beating were measured. Subsequently, the heart was stained with 1% 2,3,5‐Triphenyltetrazolium chloride (TTC) for 10 minutes at 37°C, after which it was horizontally sliced, and the infarcted area was evaluated. The infarcted area ratio was determined by dividing the infarcted area by the entire area using NIH image software.

### Statistics

Data are presented as means±SEs. Nonparametric comparisons between the 2 groups were performed using the Mann–Whitney *U* test. Kaplan–Meier survival analysis was performed to compare survival curves between the ChAT‐tg and WT mice after MI, followed by a log‐rank test. Results with *P*<0.05 were considered statistically significant.

## Results

### ChAT Expression and HIF‐1α Protein Expression

**Figure 1.**
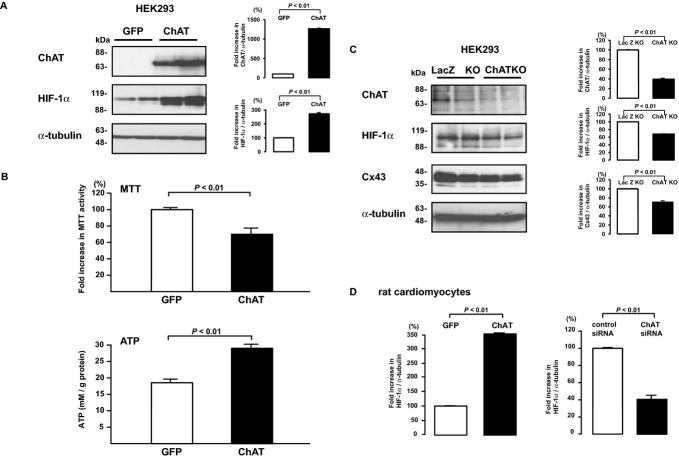
Functional analysis of ChAT using HEK293 cells and rat cardiomyocytes. **A**, HEK293 cells transfected with an expression vector for GFP or ChAT. ChAT‐transfected cells exclusively expressed HIF‐1α compared with GFP‐transfected cells. Each lane represents an independent sample. Representative data are shown from independently performed experiments (n=5 to 6). **B**, ChAT‐expressing cells showed lower MTT activity (*P*=0.0003, n=10) and higher cellular ATP content (*P*=0.0011, n=10) than did GFP‐expressing cells. **C**, HEK293 cells transfected with an expression vector for ChAT‐specific miRNA (ChAT‐KO cells). ChAT‐KO cells, already confirmed in our previous study,^1^ had lower expression of the HIF‐1α protein (*P*=0.0069) and Cx43 (*P*=0.0051) than did GFP‐expressing cells. Each lane represents an independent sample. **D**, Rat ChAT transiently transfected cardiomyocytes increased HIF‐1α expression (*P*=0.002); however, ChAT‐specific siRNA‐transfected cardiomyocytes decreased HIF‐1α expression (*P*=0.0011). Representative data are shown from independently performed experiments (n=8). ChAT indicates choline acetyltransferase; HEK, human embryonic kidney; GFP, green fluorescent protein; HIF, hypoxia‐inducible factor; MTT, 3‐(4,5‐dimethylthiazol‐2‐yl)‐2,5‐diphenyl tetrazolium bromide; miRNA, micro RNA; ChAT‐KO, choline acetyltransferase–knockout cells; Cx, connexin; siRNA, small interfering RNA.

To investigate whether enhancement of ChAT expression affects cellular energy metabolism, we initially performed 2 studies: a ChAT overexpression study and a ChAT knockdown study. The HEK293 cells transiently transfected with the *ChAT* gene expressed ChAT to a much higher degree than did the GFP‐expressing cells (*P*=0.0097; Figure 1A). In these ChAT‐expressing cells, HIF‐1α protein expression increased even under normoxic conditions (*P*=0.0069; Figure 1A), MTT activity interestingly decreased (72.5±3.8 versus 99.8±2.8, *P*=0.0003, n=10), and ATP reciprocally increased (28.2±1.8 versus 20.0±0.8 mmol/L per gram of protein, *P*=0.0011, n=10) compared with those in the GFP‐expressing cells (Figure 1B). On the other hand, HIF‐1α expression declined more in the ChAT‐KO cells than in the negative control LacZ‐KO cells (*P*=0.0069), as did total connexin 43 protein levels in ChAT‐KO cells (*P*=0.0051; Figure 1C). Likewise, ChAT‐overexpressing rat cardiomyocytes increased HIF‐1α expression (*P*=0.002; Figure 1D); in contrast, knockdown of ChAT decreased HIF‐1α expression (*P*=0.0011; Figure 1D). These results suggest that the nonneuronal cholinergic system regulated the cellular HIF‐1α protein levels (Figure 1D).

### Generation of Heart‐Specific ChAT‐tg Mice

**Figure 2.**
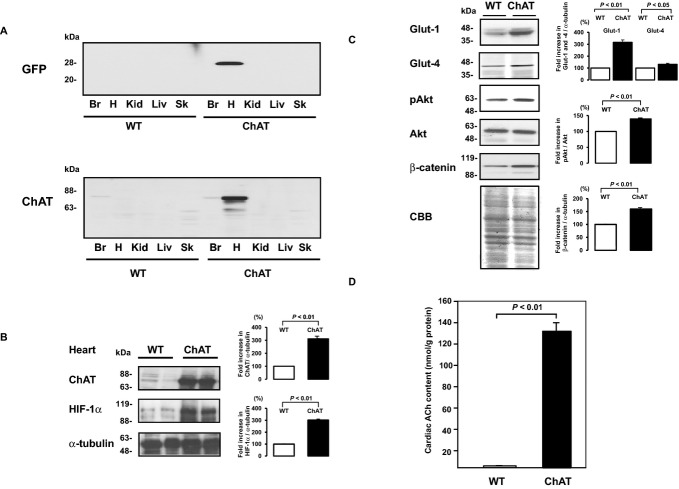
Phenotypic characteristics of the ChAT‐tg mice. **A**, Expression of GFP and ChAT in the brain (Br), heart (H), kidney (Kid), liver (Liv), and skeletal muscle (Sk) of the WT and ChAT‐tg mice. Heart‐specific expression of a transgene for GFP and ChAT was confirmed. Representative data are shown from independently performed experiments from organs of the WT and ChAT‐tg mice (n=6 each). **B**, ChAT‐tg heart had a higher level of HIF‐1α protein than did WT heart (*P*=0.0069). **C**, Expression of Glut‐1, Glut‐4, Akt, and β‐catenin. Glut‐1, Glut‐4, phosphorylated Akt, and β‐catenin were more highly expressed in ChAT‐tg heart than in WT heart. Representative data are shown from the hearts of the WT and ChAT‐tg mice (n=6). **D**, ACh level in cardiomyocytes. The ChAT‐tg cardiomyocyte had a greater level of ACh than did the WT cardiomyocyte (129.9±9.9 vs 2.3±0.2 nmol/g protein, *P*=0.0097, n=6). ChAT‐tg indicates choline acetyltransferase transgenic; GFP, green fluorescent protein; WT, wild type; Glut, glucose transporter; HIF, hypoxia‐inducible factor; CBB, coomassie brilliant blue; ACh, acetylcholine.

The ChAT‐tg mouse was developed to further investigate the biological role of the nonneuronal cardiac cholinergic system in vivo. GFP protein was detected only in the heart of the ChAT‐tg mouse; similarly, ChAT protein expression, despite endogenous expression that mainly occurred in the brain, was elevated exclusively in the heart (Figure 2A), suggesting that the ChAT‐tg mouse expresses ChAT predominantly in the heart.

Consistent with the in vitro studies (Figure 1A), the in vivo heart of the ChAT‐tg mouse expressed not only the ChAT protein but also the HIF‐1α protein to a very high degree (*P*=0.0069; Figure 2B). Glut‐1, Glut‐4, phosphorylated Akt, and β‐catenin were more expressed in the ChAT‐tg heart than in the WT heart (Figure 2C); however, total Akt protein levels were comparable. Cardiac expression of CHT1 and VAChT in the ChAT‐tg heart was not increased compared with that in the WT heart (data not shown).

ACh level in the hearts of the WT mice measured by HPLC was 2.3±0.2 nmol/g; in contrast, that of the ChAT‐tg mice was remarkably increased, to 129.9±9.9 nmol/g protein (*P*=0.0097, n=6) (Figure 2D). These results indicated that the endogenous ACh‐producing pathway was extremely activated in the ChAT‐tg cardiomyocytes.

### Hemodynamic Measurement

**Figure 3.**
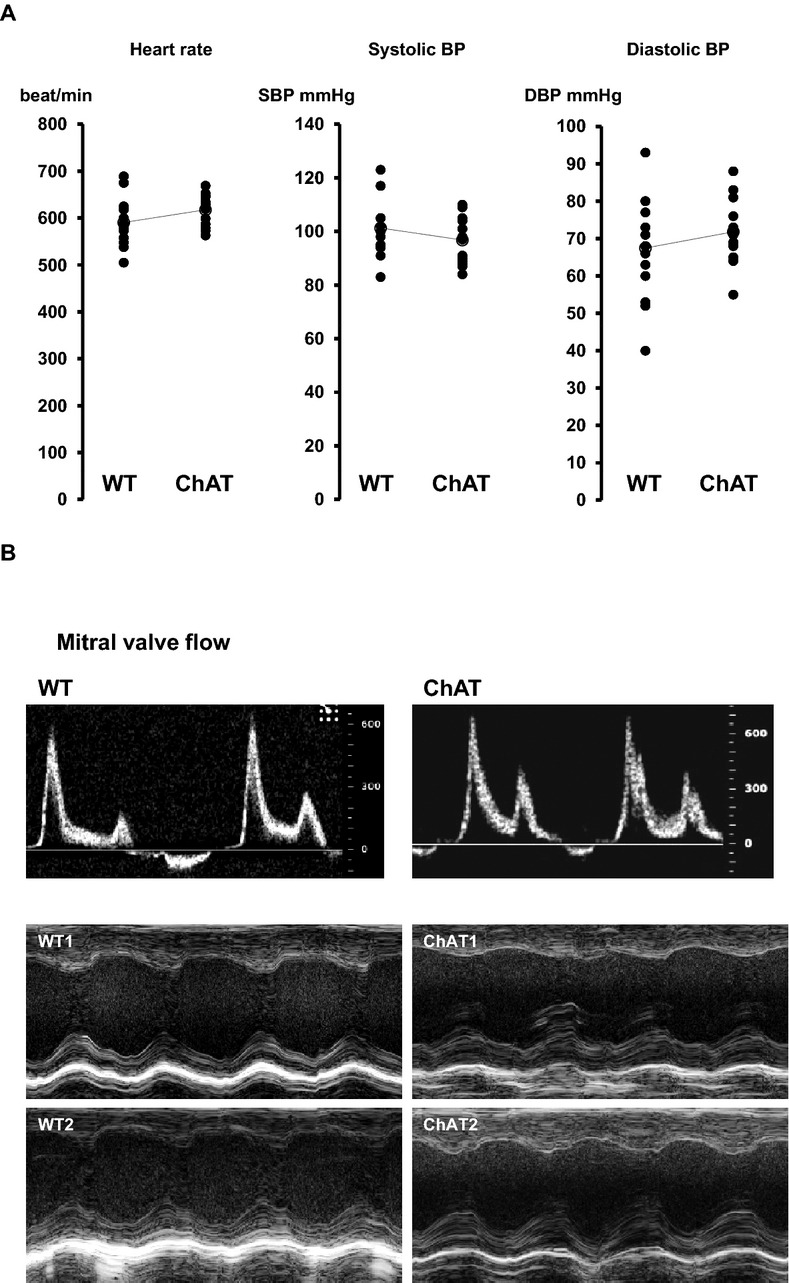
Comparison of heart rate (HR) and blood pressure (BP) and echocardiographic findings. **A**, There were no significant differences between WT and ChAT‐tg mice with regard to HR (590.5±14.4 vs 617.6±9.4 bpm, *P*=0.1811, n=13), systolic BP (101.3±2.6 vs 96.8±2.1 mm Hg, *P*=0.4592, n=15), and diastolic BP (67.5±3.4 vs 71.8±2.1 mm Hg, *P*=0.1162, n=15). **B**, Top panel: WT and ChAT‐tg hearts showed a similar pattern for mitral valve flow. Middle and bottom panels: Representative M‐mode echocardiograms of the left ventricles of the WT and ChAT‐tg mice (2 cases in each group). See Table 1 for details. WT indicates wild‐type; ChAT‐tg, choline acetyltransferase transgenic; bpm, beats per minute.

Surprisingly, HR of the ChAT‐tg mice (617.6±9.4 bpm, n=13) was not lower than that of the WT mice (590.5±14.4 bpm, n=13) (Figure 3A, *P*=0.1811), although the genotypic characteristics and the augmented ACh levels of the ChAT‐tg heart had impressed us with the prediction that the ChAT‐tg mice might show bradycardia. Likewise, SBP (96.8±2.1 mm Hg) and DBP (71.8±2.1 mm Hg) in the ChAT‐tg were comparable to SBP (101.3±2.6 mm Hg) and DBP (67.5±3.4 mm Hg)—*P*=0.4592 and *P*=0.1162, respectively—in the WT mice (Figure 3A). These results indicated that enhancement of the nonneuronal cardiac cholinergic system did not significantly affect cardiac hemodynamics.

### Echocardiographic Evaluation of Cardiac Function

Table 1 shows the summary of the parameters from the echocardiographic studies (n=9 to 10). The ChAT‐tg heart was slightly enlarged (LVDd of 4.32±0.09 mm, n=10) compared with the WT heart (3.95±0.21 mm, n=9); however, the difference was not significant. IVSTd of the ChAT‐tg heart (0.76±0.05) was comparable to that of the WT heart (0.83±0.07). EF (49.7±1.6%) and FS (25.1±1.0%) of the ChAT‐tg were slightly decreased compared with EF (53.0±1.5%) and FS (26.8±0.9%) of the WT heart; however, the differences were not statistically significant. There were no significant differences between the ChAT‐tg and WT mice in the mitral valve flow pattern, E/A ratio, or deceleration time (Figure 3B). These results indicated that overexpression of cardiac ChAT never depressed cardiac function.

**Table 1.** Echocardiographic Parameters in WT and ChAT‐tg Mice

	WT (n=9)	ChAT (n=10)	*P* Value
IVSTd, mm	0.83±0.07	0.76±0.05	0.0732
LVDd, mm	3.95±0.21	4.32±0.09	0.2780
LVDs, mm	2.89±0.17	3.25±0.28	0.0457
LVPWd, mm	0.68±0.01	0.72±0.06	0.1574
LVEDV, μL	69.3±8.8	76.2±8.6	0.0945
EF	53.0±1.5	49.7±1.6	0.0833
FS	26.8±0.9	25.1±1.0	0.0732

WT indicates wild type; ChAT‐tg, choline acetyltransferase transgenic; IVSTd, end‐diastolic interventricular septum; LVDd, left ventricular diastolic diameter; LVDs, left ventricular systolic diameter; LVPWd, left ventricular posterior wall thickness; LVEDV, left ventricular end‐diastolic volume; EF, ejection fraction; FS, fractional shortening.

### Mitochondrial Function

**Figure 4.**
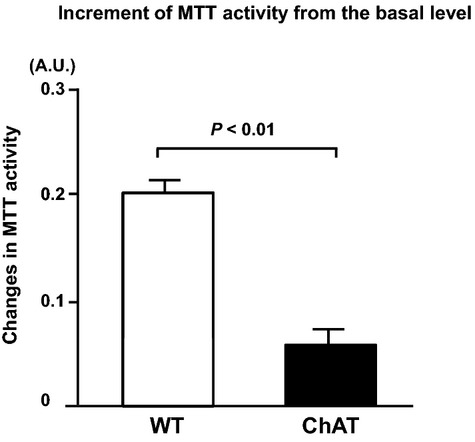
Effect of 24‐hour hypoxia on MTT reduction activity of cultured cardiomyocytes. ChAT‐tg cells had a lower baseline level of MTT activity than did WT cells, and then reoxygenation after 24‐hour hypoxia of 1% O_2_ significantly increased MTT activity of both types of cardiomyocytes; however, the incremental change in the ChAT‐tg cells was less than that in the WT cells (*P*=0.002, n=8). MTT indicates 3‐(4,5‐dimethylthiazol‐2‐yl)‐2,5‐diphenyl tetrazolium bromide; ChAT‐tg, choline acetyltransferase transgenic; WT, wild type.

Cardiomyocytes were isolated for primary culture from the ChAT‐tg and WT mice, and their MTT activities were compared on the basis of comparable cell numbers (Figure 4). It had previously been shown that MTT activity was affected by cellular energy metabolism; therefore, even with comparable cell numbers, MTT activity can decline when energy metabolism is suppressed.^1–2,12^ Under normoxic conditions, the basal MTT activity of the ChAT‐tg cardiomyocytes was significantly decreased compared with that of the WT cardiomyocytes (0.20±0.01 versus 0.22±0.01 A.U., *P*=0.0239, n=8). After 24 hours under hypoxic conditions (1% oxygen concentration) followed by normoxia, MTT activity in both types of cardiomyocytes increased from the basal levels; however, the increment in the MTT level in the ChAT‐tg cells was significantly smaller than that in the WT cells (0.06±0.02 versus 0.20±0.02 A.U., *P*=0.002, n=8; Figure 4). Under hypoxic conditions, cardiomyocytes isolated from both types of mice appeared intact; therefore, there was no difference in viable cell counts between them. These results suggest that cardiomyocytes with enhanced ChAT activity depended less on mitochondria for energy metabolism than did normal cardiomyocytes during both hypoxia and normoxia.

### Myocardial Infarction and Survival Analysis

**Figure 5.**
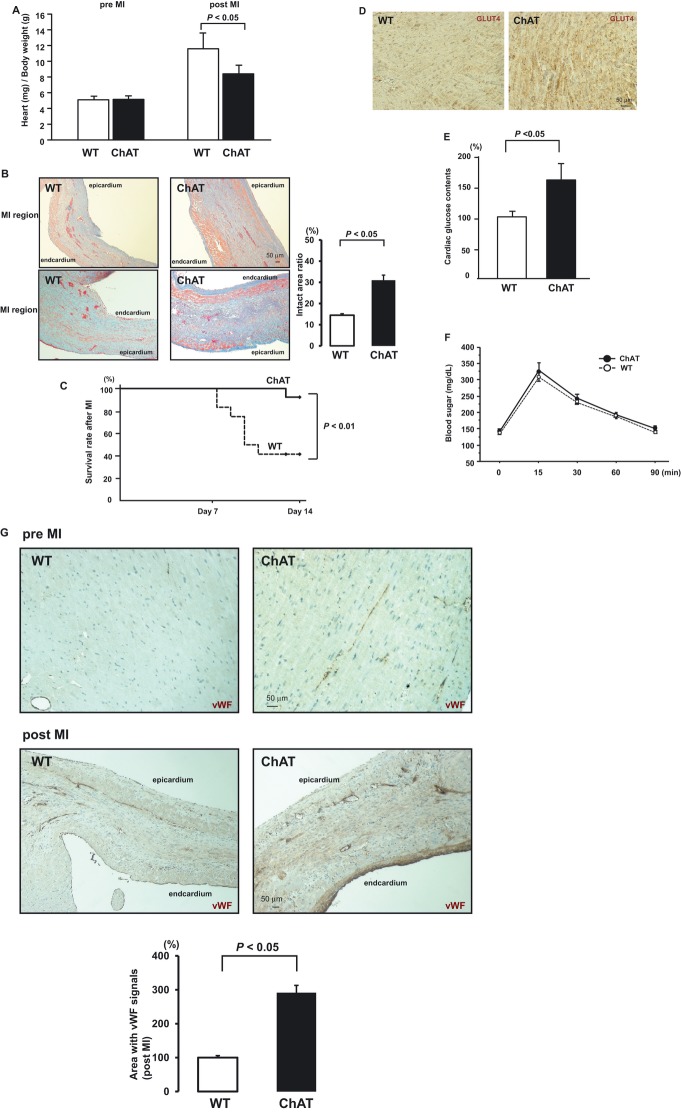
Effects of overexpression of heart‐specific ChAT on cardiac remodeling, glucose uptake, angiogenesis, and survival after myocardial infarction (MI). **A**, Heart‐to‐body weight ratio 2 weeks after MI. The weight ratio in the ChAT‐tg mice was significantly decreased compared with that in the WT mice (*P*=0.0319, n=10). **B**, Histological examination by Masson's trichrome stain. More viable myocardium (red) was observed in the layer of the ischemic left ventricular wall of the ChAT‐tg mouse than in the WT mouse, specifically in the subendothelial and subepithelial regions (*P*=0.0103, n=8); in contrast, the ischemic region of the WT infarcted heart was transmurally replaced with fibrous tissues. Scale bar=50 μm. **C**, Kaplan–Meier survival analysis of mice after MI within 14 days. ChAT‐tg mice showed a better survival rate than the WT mice after myocardial infarction (92.3% vs 41.7%, *P*=0.0048, n=13). **D**, Glut‐4 immunoreactivity in the myocardium was greater in the ChAT tg heart than in the WT mouse heart. Representative data are shown from the WT and ChAT‐tg hearts (n=6). Scale bar=50 μm. **E**, Glucose content in the ChAT tg heart was increased compared with that in the WT mouse heart (*P*=0.0379, n=5). **F**, A glucose tolerance test administered through intraperitoneal injection showed comparable blood glucose levels in the ChAT‐tg mouse and the WT mouse (n=4 each). **G**, von Willebrand factor (vWF)–positive cells were very few in the left ventricular wall of the WT sham‐operated mice, but a certain number of the cells in that of the ChAT‐tg sham‐operated mice (5G, pre‐MI ChAT). After MI, vWF‐positive cells and vascular structures more efficiently grew in the ischemic area, particularly in the ChAT‐tg heart compared with the WT heart (*P*=0.0131, n=6). Such an angiogenic image was specifically observed around and in the infarcted area. Compared with the WT infarcted heart, the ChAT‐tg infarcted heart showed an intense angiogenic response to MI (G, post‐MI ChAT). Scale bars=50 μm. ChAT‐tg indicates choline acetyltransferase transgenic; MI, myocardial infarction; WT, wild type.

To further investigate the cardiac characteristics of the ChAT‐tg mice under pathological conditions, MI was induced. Before MI, heart‐to‐body weight ratios were comparable between the ChAT‐tg and WT mice (5.06±0.29 versus 4.93±0.28 mg/g, *P*=0.2711, n=10). However, 14 days after MI, the heart‐to‐body weight ratio in the ChAT‐tg mice was significantly decreased compared with that in the WT mice (8.60±0.58 versus 11.78±0.94 mg/g, *P*=0.0319, n=10), suggesting that the ChAT‐tg heart was less susceptible to MI‐induced remodeling (Figure 5A).

MI in the WT heart caused transmural necrosis, resulting in massive myocardial loss and extensive scar formation, as shown by blue staining of Masson's trichrome stain in the left ventricle, and quite a few portions of the myocardium were left intact in the infarcted region, as shown by red staining (Figure 5B, WT). In great contrast, in the ChAT‐tg heart, more myocardial cells in the infarcted region were saved from death. The intact myocardium ratio to the infarcted area in the ChAT‐tg heart was higher than that in the WT heart (30.99±2.42% versus 14.48±0.74%, *P*=0.0103 Figure 5B). The fibrotic scar was not extended transmurally, but rather localized in the middle layer of the infarcted wall, and therefore, more viable myocardial cells were found in the subendocardial and subepicardial layers (Figure 5B, ChAT). These data suggest that the ChAT‐tg cardiomyocytes were more resistant to ischemia and could survive against MI‐induced cell death.

Survival rates between the ChAT‐tg and WT mice were compared 14 days after MI by Kaplan–Meier survival analysis (Figure 5C). Overexpression of cardiac ChAT remarkably improved survival after MI; the survival rates of the ChAT‐tg (n=13) and WT (n=13) mice were 92.3% and 41.7%, respectively (*P*=0.0048).

### Glucose Uptake

The left ventricular myocardium of the ChAT‐tg mouse had richer and more intense stains for Glut‐4 immunoreactivities than did that of the WT mouse (Figure 5D). Glut‐1 signals were hardly detected in the myocardium of both types of mice (data not shown). The glucose level in the ChAT‐tg heart was higher than that in the WT heart (158.9±21.4% versus 100.0±12.8%, *P*=0.0379, n=5; Figure 5E). Heart‐specific overexpression of ChAT accelerated cardiac glucose utilization but did not affect blood glucose level or glucose tolerance (Figure 5F).

### Angiogenesis After Myocardial Infarction

To study the angiogenic effect of the nonneuronal cardiac cholinergic system, we performed immunohistochemical experiments that used an antibody against von Willebrand factor (vWF), a marker of endothelial cells. The vWF‐positive cells were very few in the left ventricular wall of the WT sham‐operated mouse, but a certain number of the cells in that of the ChAT‐tg sham‐operated mouse expressed vWF (Figure 5G, pre‐MI). After MI, the vWF‐positive cells and vascular structures grew in the ischemic area, particularly in the ChAT‐tg heart. Such a microscopic image was specifically observed around and in the infarcted area. Compared with the WT infarcted heart, the ChAT‐tg infarcted heart showed an intense angiogenic response to MI (Figure 5G, post‐MI). The area with vWF signals was greater in the ChAT‐tg heart than in the WT heart (291.1±21.7% versus 100.0±5.9%, *P*=0.0131, n=6), suggesting that myocardium‐derived ACh specifically accelerates angiogenesis. The facilitatory effect of cardiac ACh on reactive angiogenesis would also contribute to salvage of the myocardium in the ischemic area.

### Ischemia–Reperfusion Injury

**Figure 6.**
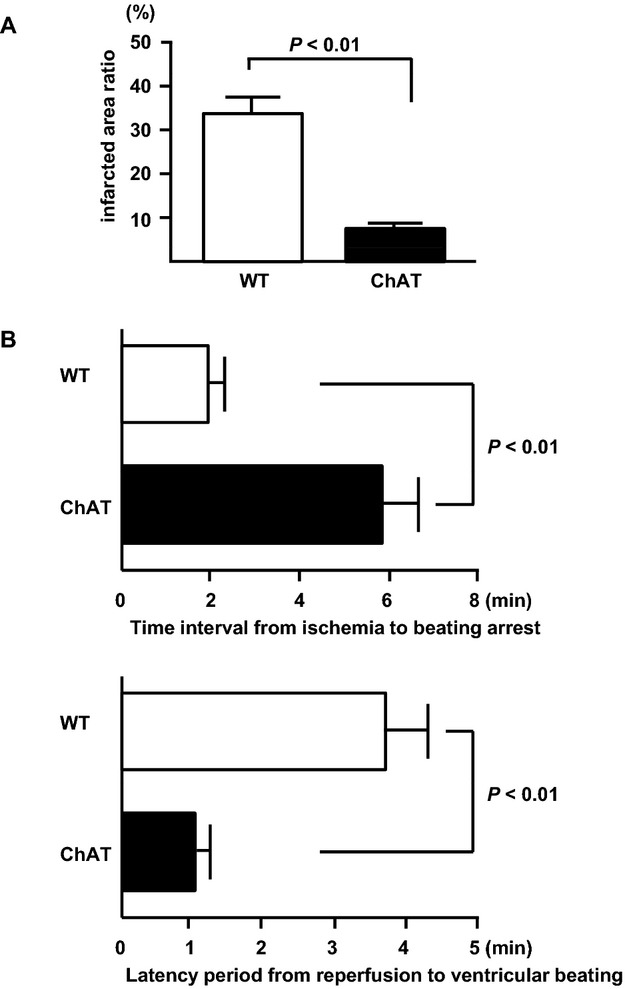
Effects of cardiac overexpression of ChAT on ischemia–reperfusion injury. **A**, Infarcted ratio of the ChAT‐tg heart evaluated by TTC stain was significantly less than that of the WT heart (*P*=0.0011, n=9). **B**, The ChAT‐tg heart had a significantly longer interval from ischemia to ventricular arrest (*P*=0.0014) and significantly shorter latency period from reperfusion to recovery of ventricular beating (*P*=0.0011) than the WT heart (n=9 each). ChAT‐tg indicates choline acetyltransferase transgenic; WT, wild type.

To investigate the effects of ChAT overexpression on acute ischemic stress, ischemia–reperfusion injury was induced using a Langendorff apparatus, which was proper to evaluate the issue independent of the neuronal regulation. The infarcted area in the WT heart was 33.2±4.9% (n=9); in contrast, the ratio in the ChAT‐tg heart was significantly suppressed, to 7.4±1.1% (versus WT, *P*=0.0011, n=9; Figure 6A). This suggests that the ChAT‐tg heart was more resistant to ischemia–reperfusion. The time interval from the onset of ischemia to beating arrest was significantly longer in the ChAT‐tg heart than that in the WT heart (5.67±0.64 versus 1.83±0.29 minutes, *P*=0.0014, n=9; Figure 6B), and the latency period from the onset of reperfusion to recovery of ventricular beating was significantly shorter in the ChAT‐tg heart than that in the WT heart (1.07±0.14 versus 3.78±0.49 minutes, *P*=0.0011, n=9; Figure 6B). These data of the time interval also support that the ChAT‐tg heart is resistant to injury and more efficiently saves the heart from energy depletion.

## Discussion

In the current study, we developed a ChAT‐tg mouse, which expressed ChAT exclusively in the heart after birth and efficiently synthesized ACh in cardiomyocytes. The comparison between ChAT‐tg and WT mice revealed new features of a nonneuronal cardiac cholinergic system in vivo: (1) cardiomyocyte‐derived ACh activates a nonhypoxic induction pathway of HIF‐1α, as supported by our previous study^6^; (2) downstream genes upregulated by HIF‐1α involve efficient glucose uptake via Glut‐4 in the heart; (3) cardiomyocyte‐derived ACh facilitates angiogenesis in the heart, as also supported by our murine hind limb ischemia model^16^; (4) cardiomyocyte‐derived ACh makes energy metabolism of cardiomyocytes less dependent on oxygen even under normoxic and postischemia–reperfusion conditions, as also suggested by our ChAT knockdown study^1^; and (5) overexpression of heart‐specific ChAT improves survival following MI induced by coronary artery ligation and ischemia–reperfusion injury.

So far, in terms of the pleiotropic action of ACh, we determined that ACh plays a crucial role in suppressing cardiac remodeling by preventing myocardium loss,^2,6,9,17^ attenuating electric remodeling,^5,18^ and facilitating angiogenesis in an ischemic hind limb model.^16^ Initially these effects were supposed to be exerted by ACh released from vagal nerve endings; however, lines of evidence have suggested that this might not be the case because of the poor innervation of the vagal nerve in the cardiac ventricles in contrast to rich adrenergic nerve endings,^19–21^ and ACh from the vagal nerve ends degraded rapidly by acetylcholinesterase, meaning that ACh could not be distributed over all the ventricles. Therefore, we recently proposed the concept that each cardiomyocyte synthesizes ACh by intrinsic component machineries, that is, a nonneuronal cardiac cholinergic system,^1^ and showed that the system was transcriptionally activated in a positive‐feedback system with muscarinic receptor agonists, because atropine, a muscarinic receptor antagonist, blunted ACh synthesis in cardiomyocytes by suppressing ChAT transcriptional activity, and ACh released by a cardiomyocyte sequentially triggered neighboring cardiomyocytes for ACh synthesis, leading to amplification of ACh synthesis in the whole heart.^1^ However, previous studies were all conducted in vitro, and no information regarding the in vivo role of the system was obtained. Therefore, to investigate the in vivo role of the cardiomyocyte ACh system, we developed ChAT‐tg mice as an animal model. As shown, the ACh level in the hearts of the ChAT‐tg mice was considerably higher than that in the WT mice; however, it was not associated with increased expression of CHT1 and VAChT in the ChAT‐tg heart compared with that in the WT heart (data not shown).

### ChAT and HIF‐1α

The ChAT‐tg mice had the following characteristics from the viewpoint of cardiac energy metabolism. First, the central phenotype of ChAT‐tg mice is an elevated HIF‐1α protein level in the heart. This suggested that the nonhypoxic induction pathway of HIF‐1α was activated by ACh in cardiomyocytes. According to our in vitro work,^6^ ACh inhibited HIF‐1α degradation, dependent on a PI3K/Akt survival pathway through a muscarinic receptor, rather than activation of HIF‐1α transcription, because a transcriptional or translational inhibitor did not inhibit ACh‐induced upregulation of HIF‐1α protein and the ACh‐induced activation of the survival pathway was inhibited by atropine. Therefore, the results suggest that HIF‐1α protein expression was sustained through ACh‐induced inhibition of its degradation in the ChAT‐tg heart.

### ChAT and Glucose Transporters

It has been known that gene expression of a glucose transporter is developmentally regulated in the heart and that Glut‐1 is predominantly expressed during the fetal period and is replaced with Glut‐4 after birth, followed by dominant expression of Glut‐4 during all of the adult period.^22–24^ Unlike the WT adult heart, the ChAT‐tg adult heart has high expression of both Glut‐1 and Glut‐4, as shown by Western blot analysis; however, only Glut‐4 was immunohistochemically detected at higher levels in the ChAT‐tg myocardium. As previously reported in several studies, Glut‐1 and Glut‐4 are predominantly upregulated by HIF‐1α.^25–26^ Taken together with the results from these reports, our results suggest that both glucose transporters were positively regulated by ACh in the ChAT‐tg heart. These phenotypes prompted us to speculate that the ChAT‐tg preferentially uses glucose through upregulated glucose transporters. Higher cardiac glucose content in the ChAT‐tg heart was consistent with this speculation.

### ChAT and Mitochondrial Function

As previously reported, an MTT reduction assay was useful for evaluation of cellular viability and mitochondrial function, that is, more oxygen consumption of the mitochondria was accompanied by higher MTT activity.^1–2,12^ Compared with the WT cardiomyocytes, the ChAT‐tg cardiomyocytes had lower MTT reduction activity under normoxic conditions, and the lower MTT reduction activity was sustained even under posthypoxia‐reoxygenation conditions, although it was increased from the baseline level, suggesting that ACh derived from the ChAT‐tg heart suppressed mitochondrial function to some extent. This characteristic phenotype was considered crucial for cardioprotection against pathological insults including hypoxia, because the ChAT‐tg cardiomyocytes showed less demand for oxygen. As demonstrated in our ischemia–reperfusion study *ex vivo*, the prolonged interval from ischemia to ventricular beating arrest in the ChAT‐tg hearts supports the concept that ACh from the ChAT‐tg cardiomyocytes efficiently suppresses energy expenditure, leading to faster recovery of ventricular beating and salvage of the heart from the injury. Taken together, these results suggest that a nonneuronal cardiac cholinergic system plays a crucial role in preventing cardiomyocytes from oxygen shortage during hypoxia and protecting the heart from hypoxia‐reoxygenation injury.

### ChAT and Angiogenesis

The ChAT‐tg showed enhanced angiogenesis in the ischemic area after MI. As previously demonstrated,^16–17^ exogenous ACh‐induced HIF‐1α transcriptionally upregulates a major angiogenic factor, vascular endothelial growth factor (VEGF). Therefore, intense and more vWF immunoreactivity‐positive signals in endothelial cells in the ChAT‐tg heart suggest that cardiomyocyte‐derived ACh accelerates angiogenesis in the ischemic heart.

### ChAT and Survival After MI

The survival rate of the ChAT‐tg mice with MI was remarkably higher than that of the WT mice. The ChAT‐tg heart utilizes more glucose and less oxygen and has more angiogenic properties than the WT heart, and therefore, the ChAT‐tg heart would be less susceptible to cardiac remodeling subsequent to MI.

This speculation is strongly supported by the findings from a recent work by Kucejova et al,^27^ which indicated that hepatic von Hippel‐Lindau (VHL) gene loss inhibited oxygen consumption, leading to higher oxygen content in the liver in a HIF‐dependent manner.^27^ These results are completely consistent with those of the current study and our previous study,^2^ indicating that an HIF‐1α‐regulated novel gene apoptosis inhibitor, which is localized in the nucleus, depressed transcription of mitochondrial transcription factor A itself and decreased oxygen consumption.

### ChAT and Cardiac Function

Initially, we had expected that overexpression of ChAT in the heart might severely depress its performance because of an increase in myocardial ACh content, but the ChAT‐tg heart was not associated with cardiovascular dysfunction.

We previously found out the existence of a nonneuronal cholinergic system in the heart,^1^ which was subsequently independently confirmed by Rana et al.^3^. They reported that aging caused a decrease in ChAT activity in the heart. Since the discovery of a nonneuronal cholinergic system in cells, it has been shown that such a local system plays a specific role in each cell independent of the parasympathetic nerve system,^28–29^ although few regulatory mechanisms for ACh release are available in nonneuronal cells, especially in cardiomyocytes. As Wessler et al^30^ reported, cholinergic communication has been established from the beginning of life in primitive organisms, and ACh signaling evolved long before the nervous system appeared and is involved in the regulation of basic cell function. This suggests that such an ACh signaling pathway and regulatory mechanisms for ACh synthesis might be conserved among varied cells, including epithelial and endothelial cells, fibroblasts, and lymphocytes. For example, the cholinergic system in keratinocytes regulates cell–cell communication in the skin,^31–32^ the system in lymphocytes is involved in modulation of the immune response,^33^ and other cell‐specific systems also have been reported including endothelial cells and bronchial epithelial cells. In contrast, biological function of the nonneuronal cholinergic system in cardiomyocytes has not been fully investigated.

## Conclusions

To our knowledge, this is the first study to clearly indicate the functional significance of a nonneuronal cardiac cholinergic system in vivo. Endogenous ACh in cardiomyocytes enhanced glucose utilization and saved oxygen consumption at baseline, and accelerated angiogenesis after MI. Overexpression of cardiac ChAT prevented cardiac remodeling and improved survival after MI or acute ischemia–reperfusion injury through these pleiotropic effects of ACh. These results suggest that cardiac ChAT is a potential therapeutic target for cardioprotection against ischemia and remodeling.
